# Inflammatory and Mucociliary Dysfunction-Based Endotypes Across the Spectrum of Chronic Airway Diseases

**DOI:** 10.1016/j.chest.2025.07.4087

**Published:** 2025-08-26

**Authors:** Erin Cant, Mathieu Bottier, Morven Shuttleworth, Jamie Stobo, Lidia Perea, Simon Finch, Merete Long, Hollian Richardson, Daniela Alferes de Lima Headley, Jeffrey T.J. Huang, Amelia Shoemark, James D. Chalmers

**Affiliations:** a Division of Respiratory Medicine and Gastroenterology, University of Dundee, Ninewells Hospital and Medical School, Dundee, United Kingdom; b Institut d’Investigacions Biomediques August Pi i Sunyer (IDIBAPS), Barcelona, Spain

**Keywords:** chronic respiratory condition, endotyping, inflammatory biomarkers, sputum properties

## Abstract

**Background:**

There is substantial overlap between features of COPD, asthma, bronchiectasis, and cystic fibrosis (CF). Each is characterized by inflammation and mucociliary dysfunction.

**Research Question:**

Is there a relationship between inflammation and mucociliary clearance in chronic respiratory conditions, and can biology, rather than disease labels, stratify patients into therapeutically relevant subtypes?

**Study Design and Methods:**

Patients were categorized according to primary disease and clinical characteristics recorded. Spontaneous sputum was collected, and inflammatory markers (neutrophil elastase and 18 cytokines), sputum properties (DNA content, mucins, rheology, dry weight), and microbiome (long-read 16S sequencing) were measured. K-means clustering was performed and parameters compared between and within disease groups. Control participants were individuals who had formerly smoked but were without respiratory disease.

**Results:**

The study included patients with asthma (n = 76), COPD (n = 91), bronchiectasis (n = 54), CF (n = 24), and control participants (n = 26). Nine cytokines (interferon-γ, IL-4, IL-5, eotaxin, eotaxin-3, thymus and activation regulated chemokine, granulocyte colony-stimulating factor, fractalkine, IL-22), neutrophil elastase, dry weight, mucins, and sputum rheology parameters were significantly different between disease groups and control participants (*P* < .05). K-means clustering identified 2 clusters defined by neutrophilic or T helper 2 (Th2) inflammation. The Th2 cluster was associated with lower sputum dry weight and DNA content and higher mucin-5B. Rheological parameters G′, G′′, and G∗ were significantly higher in the Th2 group, whereas the tangent of the loss angle δ was higher in the neutrophilic group, indicating a higher viscous to elastic ratio (*P* < .05 all comparisons). The neutrophilic cluster was associated with decreased alpha diversity (*P* = .04) and increased presence of Proteobacteria in their sputum microbiome compared with the Th2 cluster (*P* = .01). More neutrophilic inflammation was present in CF and bronchiectasis (42% of patients with COPD and 46% of patients with asthma were neutrophilic vs 78% of bronchiectasis and 87% of CF (*P* < .0001). Both clusters were present in all disease groups.

**Interpretation:**

Our results indicate that airway diseases have heterogeneous mucus properties. Patients were shown to cluster according to inflammatory endotype rather than disease label. Assessment based on disease labels may be aided by endotyping using inflammatory and mucociliary clearance biomarkers.

FOR EDITORIAL COMMENT, SEE PAGE 1275

**Take-Home Points**

**Study Question:** Is there a relationship between inflammation and mucociliary clearance in chronic respiratory conditions, and can biology, rather than disease labels, stratify patients into therapeutically relevant subtypes?

**Results:** Airway diseases exhibit heterogeneous inflammation and mucociliary clearance parameters but can be clustered into neutrophil-dominant and T helper 2-dominant subtypes independent of disease labels.

**Interpretation:** Precision medicine based on a treatable traits approach and mucociliary clearance/inflammation-based biomarkers may aid in repurposing therapies across the spectrum of airway diseases.

The airway diseases are a group of common, chronic lung diseases with high disease burden such as asthma, COPD, bronchiectasis, and cystic fibrosis (CF).^1^, ^2^, ^3^ Although these diseases may have distinct pathophysiology regarding underlying causes and disease mechanisms, they also have substantial overlap in terms of clinical features and manifestations.^1^^,^^4^, ^5^, ^6^ Overlap disorders are common, with up to 50% of patients with COPD having some features of asthma^4^, ^5^, ^6^ and patients with bronchiectasis frequently having features of COPD such as fixed airflow obstruction or features commonly associated with asthma.^1^ Increasingly, the “treatable traits” concept recognizes that inflammatory endotypes are shared between the spectrum of airway diseases and that inflammatory endotype-targeted treatments regardless of the disease label may lead to better outcomes for patients. For example, although asthma is traditionally thought of as a T helper 2 (Th2)-driven disease, subsets of patients with neutrophilic inflammation have been described.^7^^,^^8^ Conversely, in bronchiectasis, a classical neutrophilic disease, an eosinophilic endotype has been described.^9^

Mucus accumulation is evident across this spectrum of chronic lung diseases, including COPD, CF, bronchiectasis, and asthma, and patients with any of these conditions can regularly cough up sputum.^10^, ^11^, ^12^ In CF, defects in ion transport caused by mutations in the *CFTR* (cystic fibrosis transmembrane conductance regulator) gene causes mucus dehydration.^13^ Mucus properties are also altered in COPD and bronchiectasis, notably reduced hydration and increased viscosity.^10^^,^^14^^,^^15^ This characteristic of dehydrated mucus can impair mucociliary clearance and thus lead to an increased risk of pulmonary infections, inflammation, and airway remodeling or damage.^16^ The relationship between mucus properties and inflammatory endotypes could be key to understanding how patients in the spectrum of airway diseases are treated as well as essential to understanding the underlying biology. The aim of the current study was to examine mucus properties, inflammation, and clinical characteristics in patients with different airway diseases to identify shared “treatable traits.”

## Study Design and Methods

### Patients

The study included patients with any of the following diseases: asthma, COPD, bronchiectasis, and CF, as well as control participants attending clinics at a single center at Ninewells Hospital (Dundee, UK). Spontaneous sputum samples were obtained as a single timepoint from each participant. All patients had given informed consent for the use of sputum samples and data in research (approval number [TR000577]).

This study was approved by the local research ethics committee and biorepository ethical committee.

#### Inclusion Criteria

The following were inclusion criteria: spontaneously produced sputum, with a volume of at least 0.4 g from adults (> 18 years) with COPD, asthma, bronchiectasis, CF, or who had formerly smoked but were without lung disease; and provision of informed consent. Samples were obtained during clinical stability, defined as the absence of an exacerbation for a minimum of 4 weeks.

#### Exclusion Criteria

Exclusion criteria were known acute exacerbation of respiratory disease at the time of sampling and administration of CFTR modulator or corrector therapy.

#### Disease Definitions

Disease category was defined as follows: COPD was characterized by persistent respiratory symptoms and airflow limitation as per Global Initiative for Chronic Obstructive Lung Disease guidelines defined as an FEV_1_/FVC ratio < 0.70 following bronchodilator treatment^17^; asthma was defined by respiratory symptoms that vary over time and intensity, together with variable expiratory airflow limitation^18^; bronchiectasis was defined by clinical symptoms plus a bronchoarterial ratio > 1, or airways visible within 1 cm of the pleura on high-resolution CT scan^19^; and CF was defined as individuals with bi-allelic pathogenic variants in the *CFTR* gene. The control group consisted of healthy individuals who had formerly smoked but had normal lung function, as judged by FEV_1_ percent predicted and with no recent respiratory infections and no formal diagnosis of respiratory disease.

Samples were categorized according to the predominant disease as defined by review of the patients’ medical records and by a consultant respiratory physician. Overlap between conditions was noted.

#### Data Collection

The following demographic and clinical information was collected: age, sex, BMI, FEV_1_, FEV_1_ percent predicted, Medical Research Council Dyspnoea Scale score, and number of exacerbations in the previous year. Data on current medications were also collected and included use of inhaled corticosteroids, saline, macrolides, mucolytics, and DNase. Long-term use of these medications was defined as administration for at least 3 months prior to enrollment.

#### Inflammatory Markers

Sputum supernatant was prepared as described previously^20^ and stored frozen at –80 °C until use. A custom-designed Meso Scale Discovery U plex assay (Meso Scale Diagnostics) was used to measure the concentration of 17 cytokines (granulocyte-macrophage colony-stimulating factor [GM-CSF], interferon-γ [IFN-γ], IL-2, IL-4, IL-5, IL-13, eotaxin, eotaxin-3, thymus and activation regulated chemokine [TARC], granulocyte colony-stimulating factor [G-CSF], fractalkine, IL-17E/IL25, IL-22, IL-33, thymic stromal lymphopoietin [TSLP], IL-3, and eotaxin-2). In addition, IL-8 was measured by enzyme-linked immunosorbent assay (R&D Systems) and active neutrophil elastase by immunoassay (ProAxsis).

#### Sputum Properties

Mucus hydration was measured by sputum wet:dry weight ratio, calculated by dehydrating 80 μL sputum at 70 °C in a heat block for 6 hours. Duplicate samples were dehydrated, and the mean result used in analysis. A 3% ratio of wet:dry weight was considered to represent likely impaired mucociliary clearance as previously described.^16^ DNA content was measured by using the PicoGreen assay (Thermo Fisher Scientific), following the manufacturer’s instruction.

#### Rheological Properties

A 0.2 g sample of whole sputum was vortexed for 15 minutes for homogenization. The Murray sputum color scale was recorded. Rheological parameters were measured through oscillation rheology (HR-2 Discovery Series Hybrid Rheometer; TA Instruments). Briefly, 100 μL of homogenized sputum was loaded between the rheometer’s Peltier plate and a 20 mm parallel plate, which oscillate varying either frequency or stress applied to the sample. Experiments were conducted at 23 °C, and the geometer gap was set to 300 μm. A frequency sweep, which varied the oscillation frequencies from 100 rad/s to 0.1 rad/s, at a fixed stress of 1 Pa was conducted by using TRIOS software version 5.1.1.46572 (TA Instruments).

The storage modulus G′ (pascals), loss modulus G′′ (pascals), and the damping factor (tangent of the loss angle δ [tan delta]) values were extracted from the frequency sweep at the 1 Hz (6.28 rad/s) point. Absence or presence of inertia was also noted. The damping factor was calculated by using the following equation: tan *δ* = *G*′′⁄*G*′. G∗, the complex modulus, was calculated from G′ and G′′ by using the following equation: $G^{*}=\sqrt{\left[{\left(G^{\prime }\right)}^{2}+{\left(G^{\prime \prime }\right)}^{2}\right]}$.

#### Mucin Protein Quantification

Mucins were quantified by using mass spectrometry. Details of the methods, including the peptides and their corresponding isotopes selected to quantify mucin-5B (MUC5B) and mucin-5AC (MUC5AC), are shown in the Supplementary Methods.

### Microbiome Methods

DNA was extracted from 0.1 g of whole sputum using the Quick-DNA Universal Kit (Zymo) or DNeasy PowerSoil Pro Kit (Qiagen), and synthetic full-length 16S rRNA sequencing was performed by using the LoopSeq approach.^21^ Synthetic reads were processed in DADA2 and quality of reads checked using FASTQC and MultiQC in R 4.1.2 (R Foundation for Statistical Computing; December 30, 2022) prior to further analysis in R. Taxonomy was assigned against the Silva version 138.1 database. The MicrobiomeAnalyst web server was used for data analysis. Alpha diversity analyses were conducted by using the Shannon-Weiner and Chao1 indexes. Beta diversity was visualized with principal coordinate plots based on the Bray-Curtis dissimilarity index. Random forest and linear discriminant analysis effect sizes (LEfSes) were used to identify taxa that discriminated between groups.

### Statistical Analysis

Group comparisons were performed in SPSS (IBM SPSS Statistics, IBM Corporation), and data were log transformed and represented graphically in GraphPad PRISM version 9.5.1 (GraphPad Software). Comparisons were made via the Mann-Whitney *U* test or parametric equivalent as appropriate for comparisons of 2 groups. For comparisons of more than 3 groups, the Kruskal-Wallis test or 1-way analysis of variance was used; *P* < .05 was considered statistically significant. Where multiple parameters are compared between groups, adjusted *P* values were computed by using the false discovery rate (FDR) control approach of Benjamini and Hochberg.^22^

An assessment of clustering based on inflammatory profiles and sputum characteristics was performed by using the K-means algorithm; clinical characteristics and mediations were not included. Skewed distributions were log transformed to ensure all variables were on a comparable scale. Observations below the lower limit of detection of the assay were set to the assay lower limit of detection value. Cases with any missing data (assumed missing-at-random) were included using multiple imputations (10 imputations performed) with the “miculst” and “mice” packages in R version 4.2.1, based on the ideas in Basagaña et al.^23^, ^24^, ^25^ The K-means algorithm was applied to each imputed data set separately. K = 2 to 5 clusters were explored, and the optimal number of clusters (K) for each imputed data set was taken to be that which maximized the CritCF criterion.^26^ The final number of clusters was chosen to be the most common “optimal” K across all imputed data sets; similarly, the final cluster allocation for each patient was taken as the most common across all imputations. Properties were compared between the resulting clusters in the same way as for other groups (as described earlier).

## Results

### Patient Characteristics

A total of 271 patients were included in the study. The primary diagnosis was COPD (n = 91), asthma (n = 76), bronchiectasis (n = 54), and CF (n = 24). The study included 26 patients who had formerly smoked and had normal spirometry results. These patients served as control participants with no evidence of respiratory disease; they had smoked a mean ± SD of 40 ± 11.9 pack years. The characteristics of each disease group are shown in Table 1.

**Table 1:** Demographic and Clinical Characteristics of Study Patients

Characteristic	Control (n = 26)	Asthma (n = 76)	Bronchiectasis (n = 54)	CF (n = 24)	COPD (n = 91)
Age, y	58.5 (53-62.3)	64 (54-71)	70 (65-77)	29 (24-25)	67 (60-73)
Female	11 (42.3)	38 (50)	25 (46.3)	12 (50)	31 (34.1)
Smoking status					
Former	26 (100)	20 (26.3)	20 (37)	1 (4.2)	77 (84.6)
Current	0 (0)	9 (11.8)	3 (5.6)	0 (0)	12 (13.2)
Never	0 (0)	47 (61.8)	31 (57.4)	23 (95.8)	2 (2.2)
Exacerbations per year					
0	NA	10 (13.2)	5 (9.3)	3 (12.5)	43 (47.3)
1	NA	17 (22.4)	12 (22.2)	5 (20.8)	15 (16.5)
2	NA	17 (22.4)	10 (18.5)	5 (20.8)	8 (8.8)
≥ 3	NA	32 (42.1)	27 (50)	11 (45.8)	25 (27.5)
BMI, kg/m^2^	31.2 (26.5-35.4)	27 (23.7-31.9)	26.1 (22.7-31.4)	22.9 (19.3-25.0)	26.6 (22.6-31.1)
FEV_1_, L	3.0 (2.6-3.3)	1.7 (1.4-2.8)	1.58 (1.0-2.3)	1.7 (1.3-2.7)	1.27 (0.9-1.8)
FEV_1_ % predicted	102 (91.8-107.3)	73.2 (57.3-89.0)	71.2 (52.4-90.3)	60 (43.3-79.0)	52 (36.9-73)
FEV_1_/FVC	76 (72-79)	63.3 (53.7-74.0)	65.3 (53.0-72.5)	58 (45.9-69.1)	44 (34-58)
MRC Dyspnoea Scale score	1 (1-1)	2 (1.3-3)	2 (2-3)	2 (1-3)	3 (2-4)
Inhaled corticosteroids use	NA	60 (78.9)	36 (66.7)	17 (70.8)	72 (79.1)
DNase use	NA	0 (0)	0 (0)	7 (29.2)	0 (0)
Mucolytic use	NA	8 (10.5)	14 (25.9)	0 (0)	9 (9.9)
Saline use	NA	0 (0)	6 (11.1)	13 (54.2)	0 (0)
Macrolide use	NA	12 (15.8)	23 (42.6)	15 (62.5)	10 (11)

Data are presented as median (interquartile range) or No. (%). CF = cystic fibrosis; MRC = Medical Research Council; NA = not applicable.

No attempt was made to match the cohorts, as differences in characteristics were expected based on the known features of the diseases. Patients with CF were younger and had lower BMI compared with the other groups. As expected, FEV_1_ was lowest in patients with COPD. Mucoactive drugs were most frequently used in people with CF and, to a lesser extent, those with bronchiectasis.

### Comparison of Sputum Properties Between the Disease and Control Groups

**Figure 1:**
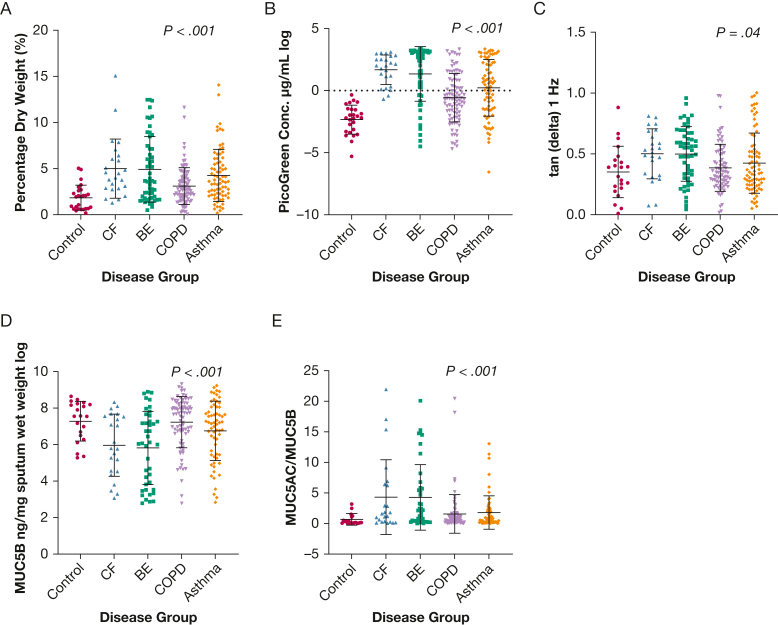
Comparison between the control, CF, BE, COPD, and asthma groups and their physical sputum parameters: percent dry weight (A), PicoGreen Conc. (log scale) (B), tangent of the loss angle δ (tan delta) at 1 Hz (C), MUC5B Conc. (log scale) (D), and ratio of MUC5AC Conc. to MUC5B Conc. (E) (means with SDs). Unadjusted Kruskal-Wallis or analysis of variance tests were used. BE = bronchiectasis; CF = cystic fibrosis; Conc. = concentration.

All disease groups had dehydrated mucus (dry weight), increased DNA content (PicoGreen assay), and increased percent MUC5AC content (MUC5AC/MUC5B ratio) compared with the control group (Fig 1). The mucin MUC5B is usually associated with respiratory health, and this was less abundant in bronchiectasis and CF sputum samples compared with asthma, COPD, and control samples. The rheological parameter tan delta, a measure of sputum viscosity and elasticity, was increased in the CF and bronchiectasis groups compared with the control, asthma, and COPD groups.

There were no significant differences between the disease groups and the control group in the rheological properties $G^{\prime }$ (1 Hz), *G*′′ (1 Hz), and G∗ (1 Hz) or the concentration of MUC5AC (e-Table 1).

Three percent dry weight has been identified as the mucus dehydration associated with impeding mucociliary clearance and mucus status. Patients with a dry weight associated with mucus impedance was identified in all disease groups. A dry weight > 3% was most common in CF sputum (70.8%) and asthma sputum (60.5%), bronchiectasis (55.6%), and COPD (42.9%) compared with control participants (in whom only 19.2% of the group had > 3% dry weight).

### Comparison of Sputum Inflammatory Markers Between the Disease and Control Groups

**Figure 2:**
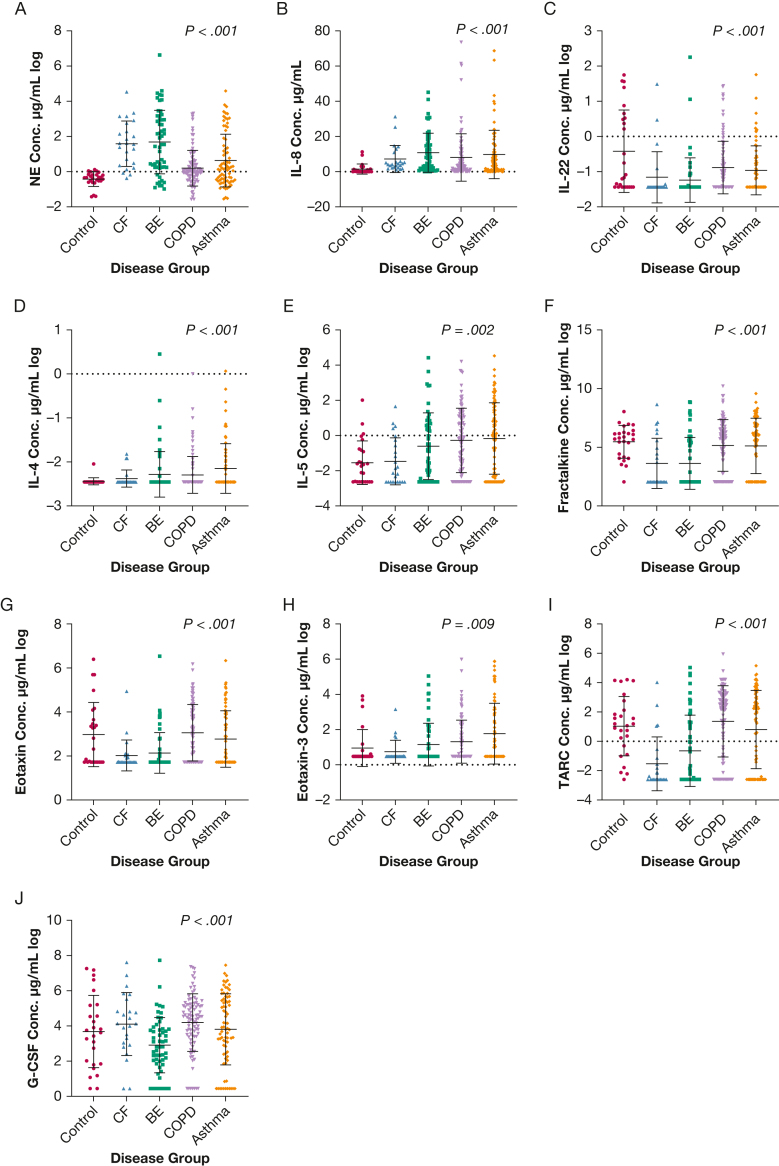
A-J, Comparison between the control, CF, BE, COPD, and asthma groups and their sputum cytokine concentrations: NE (log scale) (A), IL-8 (B), IL-22 (log scale) (C), IL-4 (log scale) (D), IL-5 (log scale) (E), fractalkine (log scale) (F), eotaxin (log scale) (G), eotaxin-3 (log scale) (H), TARC (log scale) (I), and G-CSF (log scale) (J) (means with SDs). Unadjusted Kruskal-Wallis or analysis of variance tests were used. BE = bronchiectasis; CF = cystic fibrosis; Conc. = concentration; G-CSF = granulocyte colony-stimulating factor; NE = neutrophil elastase; TARC = thymus and activation-regulated chemokine.

Ten inflammatory markers were significantly different between the disease groups and control groups (Fig 2). Neutrophil elastase was highest in patients with CF and bronchiectasis and was very low in control participants. Patients with COPD and asthma had the lowest levels, but levels in both these patient groups were still significantly higher than levels in control participants. IL-8 levels were highest in patients with bronchiectasis and again were higher in all disease groups compared with the control group. In contrast, the predominantly Th2-induced cytokine IL-4 was significantly higher in the asthma group compared with the other disease groups and was not significantly different between patients with CF and control participants (unadjusted *P* = .1). As anticipated, the eosinophil-related cytokine IL-5 had the highest mean values in asthma, second highest in COPD, and equivalent levels to control participants in the bronchiectasis and CF groups.

This evidence of increased eosinophilic airway disease in patients with asthma and COPD was seen also in the eotaxins, the eosinophil chemotactic mediators. Eotaxin-1 was highest in the COPD, asthma, and control groups and low in the bronchiectasis and CF groups. Eotaxin-3 was highest in patients with asthma and lowest in those with CF. TARC levels were highest in the asthma, COPD, and control groups and lowest in the CF and bronchiectasis groups; a similar pattern observed for fractalkine. Interestingly, G-CSF showed a disease-specific signature with significantly lower levels in bronchiectasis and relatively similar levels in the other 4 groups. Finally, IL-22 was highest in the control group and lowest in the CF and bronchiectasis groups. There were no significant differences between the groups in IL-17A, GM-CSF, IFN-γ, IL-2, IL-13, IL17E, or IL-33.

In summary, the cytokine analysis suggested very similar profiles between CF and bronchiectasis while asthma in particular exhibited a different pattern characterized by Th2 inflammation.

### Patient Stratification by Biology Rather Than Disease Label: Characteristics of 2 Clusters From K-Means Algorithm With Multiple Imputation

To explore the hypothesis that biology, rather than disease labels, may stratify patients into therapeutically relevant subtypes, the sputum and inflammatory marker data were used to perform an unsupervised K-means cluster analysis. Information on disease labels was not included in the analysis.

**Figure 3:**
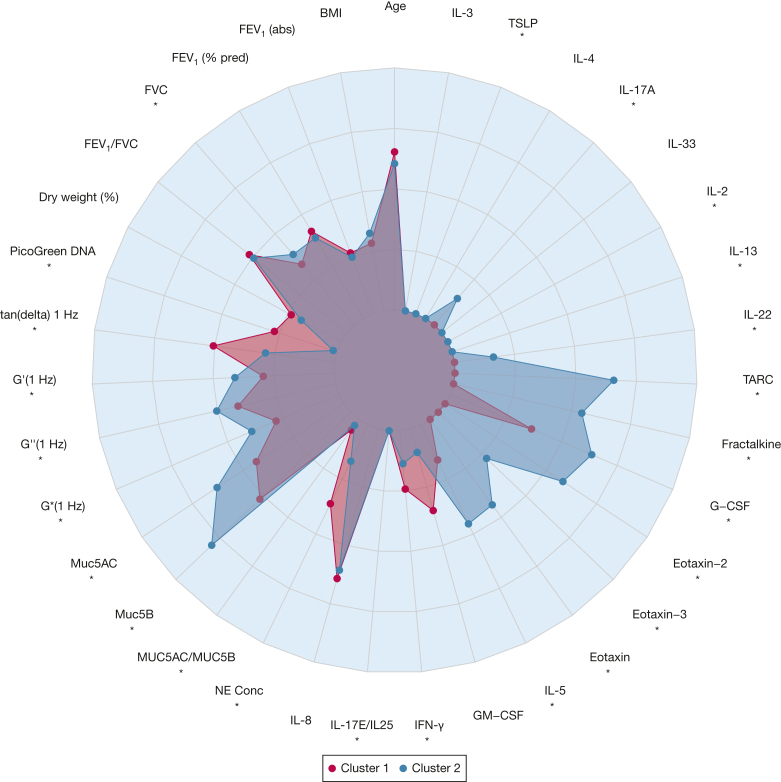
Radar (spider) plot showing median parameter values (relative to parameter range) for each cluster. Asterisks indicate significant differences between the two clusters (false discovery rate-adjusted *P* < .05). abs = absolute; Conc = concentration; G-CSF = granulocyte colony-stimulating factor; GM-CSF = granulocyte-macrophage colony-stimulating factor; IFN-γ = interferon-γ; mucin-5AC = MUC5AC; MUC5B = mucin-5B; NE = neutrophil elastase; pred = predicted; TARC = thymus and activation regulated chemokine; TSLP = thymic stromal lymphopoietin.

Multiple imputation of missing data for G′ (1 Hz), G′′ (1 Hz), tan (delta) 1 Hz, G∗ (1 Hz), MUC5B, and MUC5AC was performed. Two clusters appeared to be optimal, maximizing the CritCF criterion in all imputed data sets. Cluster 1 was associated with more neutrophilic-driven disease biomarkers (neutrophil elastase, IL-2, and IFN-γ; n = 139). Cluster 2 was associated with Th-2-driven disease biomarkers (IL-5, IL-4, IL-13, IL-22, eotaxin, eotaxin-2, eotaxin-3, fractalkine, G-CSF, TARC, and TSLP; n = 106). Comparisons are shown in Figure 3. The neutrophilic cluster was associated with higher sputum dry weight and DNA content. The Th-2 cluster was associated with increased MUC5B. Rheological parameters G′, G′′, and G∗ were significantly higher in the Th-2 cluster while tan (delta) was higher in the neutrophilic cluster (FDR-adjusted *P* < .05 for all comparisons) (e-Table 1, Fig 3).

Strikingly, when comparing the clinical characteristics, there were no significant differences between the two clusters (seen visually in Figure 3 and quantitively in Table 2). Patients with CF and bronchiectasis were more likely to be in cluster 1, whereas patients with COPD and asthma were evenly distributed between the 2 clusters. There was no significant difference between the percentage of patients with high blood eosinophil counts (> 300 cells/μL) comparing cluster 1 (25.7%) vs cluster 2 (33%).

**Table 2:** Clinical Characteristics and Diseases of Participants Compared Between 2 Clusters Based on Sputum Physical Properties and Inflammatory Markers

	Cluster 1 (Neutrophilic; n = 139)	Cluster 2 (Th 2; n = 106)
Condition (*P* = 4.07 × 10^-6^)		
Asthma	36 (25.9%)	40 (37.8%)
BE	42 (30.2%)	12 (11.3%)
CF	21 (15.1%)	3 (2.8%)
COPD	40 (28.8%)	51 (48.1%)
MRC Dyspnoea score (*P* = .520)		
1	28 (20.1%)	20 (18.9%)
2	36 (25.9%)	34 (32.0%)
3	39 (28.1%)	29 (27.4%)
4	26 (18.7%)	18 (17.0%)
5	10 (7.2%)	5 (4.7%)
No. of exacerbations in the past year (*P* = .729)		
Minimum	0	0
Q1	1	0
Median	2	2
Mean	2.223	2.302
Q3	3	3
Maximum	12	13
No. of severe exacerbations (hospitalizations) (*P* = .735)		
Minimum	0	0
Q1	0	0
Median	0	0
Mean	0.259	0.2453
Q3	0	0
Maximum	4	3

Categorical data are presented as No. of participants (%). Continuous data are presented as minimum, lower quartile (Q1), median, mean, upper quartile (Q3), and maximum. Unadjusted 2-sided *P* values comparing distribution of characteristics between clusters are provided. BE = bronchiectasis; CF = cystic fibrosis; Th2 = T helper 2.

### Comparison of Microbiome Characteristics Between Clusters

**Figure 4:**
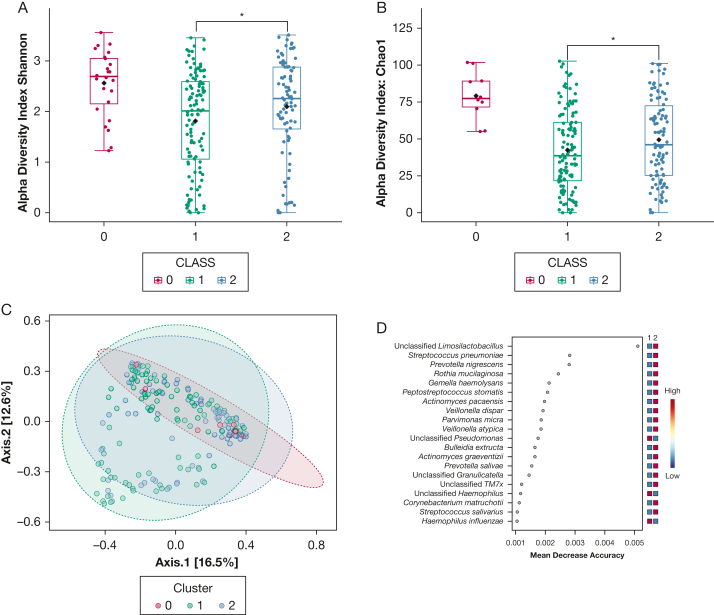
Comparisons of the microbiome characteristics between control group (0), cluster 1 group (neutrophilic group) (1), and cluster 2 group (Th2) (2). The following were assessed: A, Alpha diversity index: Shannon-Weiner (Shannon); B, Alpha diversity index: Chao1; and C, Beta diversity. D, Forrest plot showing microbiota driving the differences between cluster 1 group and cluster 2 group. Unadjusted *P* values were used for the comparisons between alpha and beta diversity of clusters. Asterisks indicate *P* < .05.

The alpha diversity and beta diversity were compared between the control group (0), cluster 1 (1), and cluster 2 (2). The alpha diversity measured by using the Shannon-Weiner diversity index and the Chao1 diversity index in the disease groups (cluster 1 and cluster 2) was decreased compared with the control group (*P* = .002). The cluster 1 (1) has reduced alpha diversity compared with cluster 2 (2) (unadjusted *P* = 0.04). Comparisons are shown in Figures 4A and 4B. The 3 groups (control, cluster 1, and cluster 2) also have distinct beta diversity (unadjusted *P* < .0001 by permutational multivariate analysis of variance) (Fig 4C).

As indicated in Figure 4D, random forest analysis showed a higher abundance of *Streptococcus pneumoniae* and commensal taxa such as *Prevotella* and *Rothia mucilaginosa* in cluster 2 (Th2 inflammation). *Pseudomonas* and *Haemophilus* were enriched in cluster 1. LEfSe revealed that this difference between the groups was driven by a dominance of Proteobacteria such as *Pseudomonas aeruginosa* (linear discriminant analysis [LDA] effect size score = 2.61; FDR-adjusted *P* = .0041), and *Haemophilus parainfluenzae* (LDA score = 2.08; FDR-adjusted *P* = .012) in the neutrophilic group (1). The Th2 group (2) was more dominated by *Streptococcus* (LDA score = 2.93; FDR-adjusted *P* ≤ .0001), *R mucilaginosa* (LDA score = 2.68; FDR adjusted *P* = .0001), and *Prevotella melaninogenica* (LDA score = 2.36; FDR-adjusted *P* = .031).

## Discussion

The current study found considerable overlap in sputum biophysical and inflammatory properties between the different common airway diseases. Traditional teaching is that CF and bronchiectasis (and to some extent COPD) are highly neutrophilic, whereas asthma is eosinophilic. This has been clearly refuted in asthma in which subtypes of non-eosinophilic, atopic, T helper 17, and pauci-inflammatory asthma have all been described distinct from classical allergic eosinophilic asthma.^27^^,^^28^ The current study data suggest there are also large differences within the other airway diseases.

For practical reasons, as it influences treatment with anti-Th2 therapies such as corticosteroids and biologics, asthma has been considered in some guidance as Th2 and non-Th2, indicating 2 “clusters.”^8^ It is therefore striking that this cluster analysis, which was unsupervised/unbiased, identified 2 clusters of patients that separate (based on the known biological function of the markers) into Th2 and non-Th2 (neutrophilic). This scenario suggests that the endotypes seen in asthma may be observed to an extent across all airway diseases. The cluster 2 had raised levels of IL-5, the classic eosinophil-related cytokine, as well as eotaxins and other Th2 markers. Neutrophil elastase, a marker associated with airway inflammation and infection,^29^ was elevated in cluster 1. It is notable that many patients were treated with inhaled corticosteroids regardless of inflammatory endotype, suggesting these drugs may be overused in inflammatory airway diseases.

The Th2 and neutrophilic clusters had a distinct microbiome. Investigating this further suggested that cluster 1 had lower diversity and was more abundant in Proteobacteria such as *Pseudomonas*, and *Haemophilus*; cluster 2 had higher levels of *Streptococcus, Rothia,* and *Prevotella.* These findings suggest that cluster 1 could be associated with worse clinical outcomes, as high abundance of Proteobacteria has been consistently shown to be associated with exacerbations and mortality.^30^

Intriguingly, the mucus rheology was significantly different between the two groups with higher G′, G′′, and G∗ in cluster 2 and higher tan delta in cluster 1. Both groups had similar levels of mucus dehydration. MUC5B was higher in cluster 2, and DNA was much higher in cluster 1. Our data suggest that although both groups have impaired mucociliary clearance, this impairment arises through 2 different mechanisms. G′ is a measure of the solid nature (elasticity) of the sample, and G′′ is the viscous nature (plasticity) of the sample. Tan (delta) is G′′/G′ and therefore a measure of the liquid/solid ratio of the sample. G∗ is also calculated from G′, and G′′ is a measure of the rigidity of the sample. This suggests that in cluster 2, where G′, G′′, and G∗ are higher, these patients have more rigid sputum. The higher tan (delta) in the neutrophilic group suggests a higher viscous-to-elastic ratio.

These findings may be useful to guide future studies aiming to alter mucus properties in airway diseases. Given the association between inflammation and mucus properties, it would be interesting to observe the effect of anti-inflammatory treatments on mucus properties as well as mucoactive drugs and whether these have varying effects in patients from different clusters. This endotyping approach could be used to treat patients depending on their biologic characteristics regardless of the disease label. Eosinophilic inflammation is an example of cross disease endotype-driven drug repurposing exemplified by recent positive studies of dupilumab in COPD, as an example.^31^ Because mucus rheology and airway cytokines are currently not available in a clinical setting, more accessible biomarkers of inflammatory endotypes across airway diseases are needed.

Important limitations of the current study should be acknowledged. This was a single-center study and, although large overall for a study of this kind, the individual sample size for some groups was small. The high volume of sputum required to complete all analyses may bias the study toward patients with more severe disease; similarly, the reliance on spontaneous sputum will also introduce some bias because not all patients, particularly those with asthma and COPD, will produce spontaneous sputum. We were also limited by available sputum volumes regarding the number of assessments that could be performed. These results apply to patients who can produce a good volume of sputum, but we cannot comment on what results would be observed in patients with lower volume sputum production. A confirmatory study using bronchoscopy would be the only way to definitively address this issue.

There is considerable overlap between conditions, and although using the primary condition as the item for analysis is consistent with what is done in randomized trials, it has important limitations. Patients with asthma and COPD were not consistently scanned by CT imaging, and thus some may have undiagnosed bronchiectasis. Misdiagnosis and overlapping diagnoses are common and can only be partially accounted for in analysis. Nevertheless, this clinical reality emphasises the importance of having a precision medicine approach based on biology for patients with complex airway diseases. We used a control population of “healthy” individuals who had formerly smoked but had normal spirometry results. Although they did not have airway disease, these individuals with chronic former smoking habits do not have healthy airways, and this factor is reflected in a small percentage of patients who have > 3% dry weight sputum. A truly healthy comparator population for a spontaneous sputum study is not possible. Patient treatments such as inhaled corticosteroids or mucoactive drugs may influence inflammatory and rheological parameters, and it is not possible within this design to withdraw such treatments to eliminate this bias.

There have been some previous studies of rheology, particularly in CF and bronchiectasis. Ramsey et al^10^ also found that the percent dry solids (percent dry weight) were significantly higher in patients with bronchiectasis compared with healthy individuals. The current study also found a significant difference between the percent dry weight of individuals with bronchiectasis compared with the control individuals; this was also true in the other respiratory conditions compared with the control individuals. Concerning the rheological parameters, Ramsey et al^10^ found that the viscous modulus, G′′, and the elastic modulus, G′, were increased in patients with bronchiectasis compared with healthy control participants. In comparison, we did not find that the viscous or elastic modulus was significantly different in bronchiectasis. This again could be due to a smaller number of individuals included in the study compared with the study by Ramsey et al^10^ or the use of a control population consisting of individuals who formerly smoked.

## Interpretation

This study found that mucus dehydration is associated with inflammation and disease severity across airway diseases. Two clusters of airway biology were observed associated with neutrophilic and Th2 inflammation. Both clusters show evidence of mucus dehydration, suggesting that therapies targeting mucociliary clearance may have broad applicability across airway diseases. This hypothesis should be tested in future randomized trials.

## Funding/Support

This study was funded by an investigator-initiated research grant from 10.13039/100004336Novartis AG. A. S. and J. D. C. are funded by Asthma and Lung UK.

## Financial/Nonfinancial Disclosures

The authors have reported to *CHEST* the following: J. D. C. reports grants or contracts from AstraZeneca, Genentech, Gilead Sciences, GlaxoSmithKline, Insmed, Grifols, Novartis, and Boehringer Ingelheim; and consulting fees from AstraZeneca, Chiesi, GlaxoSmithKline, Insmed, Grifols, Novartis, Boehringer Ingelheim, Pfizer, Janssen, Antabio, and Zambon. A. S. reports grants or contracts from AstraZeneca and LifeArc; consulting fees from Spirovant, Translate Bio and ReCode Therapeutics; and payments and honoraria from Translate Bio, Ethris and Insmed. M. B. reports consulting fees from ReCode Therapeutics. None declared (E. C., M. S., J. S., L. P., S. F., M. L., H. R., D. A. d. L. H., J. T. J. H.).

## Supplementary Data

e-Online Data

e-Online Data 1
