# Supplementary material for: Inflammatory and Mucociliary Dysfunction-Based Endotypes Across the Spectrum of Chronic Airway Diseases
Source: Chest. 2025 Aug 26;168(6):1317–29. doi: 10.1016/j.chest.2025.07.4087 (PMC12833481; doi:10.1016/j.chest.2025.07.4087)
Supplement: e-Online Data [file mmc1.docx]

| **Parameter** | **2-sided p-value (unadjusted)** | **2-sided p-value**  **(FDR adjusted)** |
| --- | --- | --- |
| GM-CSF ^a^ | 0.481 | 0.554 |
| NE Conc ^a^ | 1.03E-17 | 4.91E-17 |
| IL2 ^b^ | 0.000761 | 0.00161 |
| IL4 ^b^ | 0.0950 | 0.124 |
| IL8 ^b^ | 0.717 | 0.758 |
| Eotaxin ^b^ | 4.34E-44 | 5.50E-43 |
| IFN-γ ^b^ | 0.0274 | 0.0453 |
| IL17A ^b^ | 0.00206 | 0.00412 |
| IL13 ^b^ | 0.0450 | 0.0683 |
| IL17E/IL25 ^b^ | 0.0471 | 0.0688 |
| IL22 ^b^ | 2.24E-22 | 1.42E-21 |
| IL3 ^b^ | 0.446 | 0.530 |
| IL33 ^b^ | 0.831 | 0.831 |
| IL5 ^b^ | 1.09E-08 | 3.45E-08 |
| TSLP ^b^ | 0.00453 | 0.00820 |
| Eotaxin-2 ^b^ | 2.26E-46 | 4.29E-45 |
| Eotaxin-3 ^b^ | 1.21E-25 | 9.20E-25 |
| Fractalkine ^b^ | 3.95E-39 | 3.75E-38 |
| G-CSF ^b^ | 3.09E-21 | 1.68E-20 |
| TARC ^b^ | 2.83E-53 | 1.07E-51 |
| Dry weight % ^a^ | 0.0916 | 0.124 |
| Nanodrop Conc ^a^ | 0.000508 | 0.00114 |
| PicoGreen DNA ^a^ | 1.22E-14 | 4.63E-14 |
| Pre PCR Qubit conc ^a^ | 0.0102 | 0.0175 |
| G’(1Hz) ^a^ | 1.42E-06 | 3.86E-06 |
| G’’(1Hz) ^a^ | 0.000233 | 0.000553 |
| G*(1Hz) ^a^ | 2.77E-06 | 7.01E-06 |
| Muc5AC ^a^ | 1.24E-11 | 4.29E-11 |
| Tan(delta) (1Hz) ^a^ | 2.67E-07 | 7.80E-07 |
| Muc5AC/Muc5B ^a^ | 0.00338 | 0.00642 |
| Muc5B ^a^ | 6.19E-15 | 2.61E-14 |

**eTable 1:** Comparison of sputum parameters between the two clusters.

^a^ Clusters compared using a Mann-Whitney test.

^b^ Clusters compared using a Peto & Peto modification of the Gehan-Wilcoxon test for left-censored data.
