# Supplementary material for: Inflammatory and Mucociliary Dysfunction-Based Endotypes Across the Spectrum of Chronic Airway Diseases
Source: Chest. 2025 Aug 26;168(6):1317–29. doi: 10.1016/j.chest.2025.07.4087 (PMC12833481; doi:10.1016/j.chest.2025.07.4087)
Supplement: e-Online Data 1 [file mmc2.docx]

**Supplementary Methods**

**Mucin quantification by mass spectrometry.**

Sputum was solubilised and alkylated using DTT (50nM) and N-ethylmaleimide (15mM). The samples were digested into peptides using trypsin/lys-C (1:100, Promega) for 18 hours at 37°C on a shaking platform. Four heavy labelled peptide internal standards from MUC5AC and MUC5B (see below, purity >99%, Peptide Synthetics, UK) from two different regions of MUC5B or MUC5AC were spiked into sputum digests at final concentrations of 20 ng/ml.

| **MUC5B** | **MUC5AC** |
| --- | --- |
| LTDPNSAFSR | GTDSGDFDTLENLR |
| LTPLQFGNLQK | LYPAGSTIYR |
| LTDPNSAFS[Arg(13C6, 15N4)] | GTDSGDFDTLENL[Arg(13C6, 15N4)] |
| LTPLQFGNLQ[Lys(13C6; 15N2)] | LYPAGSTIY[Arg(13C6, 15N4)] |

e-table 2: Peptides and their corresponding isotopes selected to quantify MUC5B and MUC5AC.

Samples were acidified in 0.1% formic acid before being subjected to a solid phase extraction using a Stagetip loaded with 4 layers C18 extraction disks (3M, Empore). Peptides were eluted with 50% acetonitrile were vacuum dried and reconstituted in 20 μL 2% acetonitrile, 0.1% formic acid in MS grade water. digested sample was injected to an Acclaim PepMap 100 column (1,000 μm, length = 15 cm, 3 μm, 100 Å, ThermoFisher) fit to a Dionex 3000 LC system linked to a triple quadrupole mass spectrometer (Quantum Ultra, Thermo Scientific) using an IonMax Interface.

Tryptic peptides were injected to an Acclaim PepMap 100 column (1,000 μm, length = 15 cm, 3 μm, 100 Å, ThermoFisher) fit to a Dionex 3000 LC system linked to a triple 25 quadrupole mass spectrometer (Quantum Ultra, Thermo Scientific) using an IonMax interface.
